# Health Literacy from A to Z: Practical Ways to Communicate Your Health Message

**Published:** 2005-03-15

**Authors:** Darren A. DeWalt

**Affiliations:** Division of General Internal Medicine, University of North Carolina School of Medicine, Chapel Hill, NC

**Figure:**
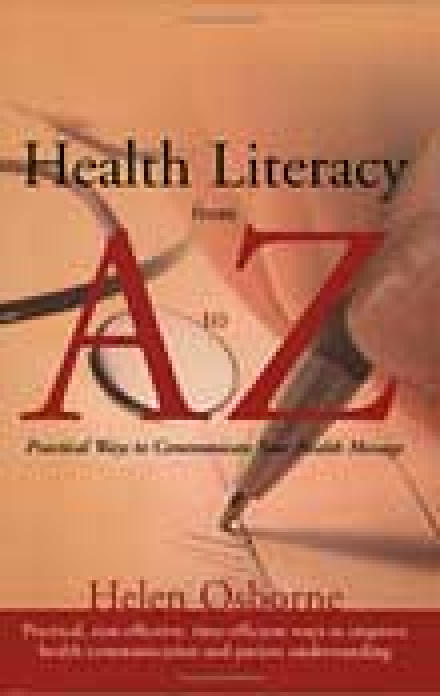


In *Health Literacy from A to Z: Practical Ways to Communicate Your Health Message*, Helen Osborne takes us into the world of a health literacy consultant and the work she does to improve health communication. Over the last 10 years, health literacy has received increasing attention from researchers, policy makers, and practitioners, and Osborne has distinguished herself as one of the leading consultants in this area. As she defines it, health literacy is "a shared responsibility in which patients and providers each must communicate in ways the other can understand." To those who accept this responsibility and are trying to communicate health messages more clearly, the need for health literacy consultants has become apparent. In this book, Helen Osborne offers an expert's guide to strategies and techniques for improving health communication.

Osborne offers a different perspective than the classic book *Teaching Patients with Low Literacy Skills* by Doak, Doak, and Root (1). Although Osborne does give practical advice on how to overcome the barrier of low literacy, much of her book focuses on health communication strategies for use with patients of all reading levels. In keeping with her broader definition of health literacy, Osborne's book is more about improving health communication generally than it is about overcoming the barrier of low reading skills.

Osborne has formatted the book in a unique and creative way. She organized the chapters alphabetically (hence the "A to Z" in the title), and each can stand alone. Each chapter contains a section called "Starting Points," which is an overview of the topic, followed by "Strategies, Ideas, and Suggestions," a section that gives practical tips and action items on the topic for the chapter. Each chapter is short and can usually be read in less than 10 minutes. As such, this book offers a brief overview of the included topics. Most readers will want to use this book as an introduction and supplement it with other literature. Osborne offers a few of her favorite references at the end of each chapter to help the reader get started.

*Health Literacy from A to Z *covers a broad scope of topics, from "plain language" and document formatting to e-mail communication, Web site design, and touchscreen technology. Osborne also covers difficult topics such as shared decision making, risk communication, and dealing with barriers such as blindness or hearing difficulties. She even includes a chapter on Feng Shui and how this ancient Chinese art can improve health communication. 

Osborne gives practical advice with a conversational tone. As I was reading the book, I felt like I was having a series of hallway conversations with a trusted colleague. Her style is accessible and easy to follow. For those who demand strict empirical evidence to adopt an idea, this book does not offer it. Osborne lists references at the end of each chapter, but she is limited by the dearth of outcomes-based research in this area (2). However, she provides the evidence of experience and consensus from years of work in this area and advice from several of her colleagues. For those who are ready to improve health communication in their institution or individual practice, this book gives excellent ideas for immediate use. It will get you started down the road to clear health communication. Had this book been available, I would have recommended it countless times over the last few years to individuals who have asked me about improving their health message.

*Health Literacy from A to Z *complements other books and reports published in 2004 on the topic of health literacy (3-5). These have focused more on the research and policy implications than on practical strategies for a variety of health communication efforts. Simply reading Osborne's book will not make you an expert on health literacy, but it will likely make you a more effective communicator in everyday practice. It will be a helpful book for medical students interested in honing these skills.

Health care providers, including nurses, pharmacists, physician assistants, administrative assistants, health educators, administrators, and physicians interested in improving health communication will enjoy this book. Specifically, it will benefit those charged with improving their organization's health message or advocating for health education. Osborne has done us a service by giving us her insights from a career in health literacy consulting.
